# What Is “Natural”? Consumer Responses to Selected Ingredients

**DOI:** 10.3390/foods7040065

**Published:** 2018-04-23

**Authors:** Edgar Chambers, Edgar Chambers, Mauricio Castro

**Affiliations:** Center for Sensory Analysis and Consumer Behavior, Kansas State University, Manhattan, KS 66506, USA; echamber@ksu.edu (E.C.); mauriciod@ksu.edu (M.C.)

**Keywords:** natural, ingredient, consumer, perception

## Abstract

Interest in “natural” food has grown enormously over the last decade. Because the United States government has not set a legal definition for the term “natural”, customers have formed their own sensory perceptions and opinions on what constitutes natural. In this study, we examined 20 ingredients to determine what consumers consider to be natural. Using a national database, 630 consumers were sampled (50% male and 50% female) online, and the results were analyzed using percentages and chi-square tests. No ingredient was considered natural by more than 69% of respondents. We found evidence that familiarity may play a major role in consumers’ determination of naturalness. We also found evidence that chemical sounding names and the age of the consumer have an effect on whether an ingredient and potentially a food is considered natural. Interestingly, a preference towards selecting GMO (genetically modified organisms) foods had no significant impact on perceptions of natural.

## 1. Introduction

Humans have an instinctual desire for natural products and processes [1]. Consequently, the natural food market has grown substantially in recent decades. The Nielsen Global Health and Wellness Survey [2] found that in addition to eating fewer fats and sugary sweets, 60% of those dieting in North America were eating more “natural” and “fresh” foods, while 56% of Europeans and 68% of Latin Americans said the same. Customers said that the three “most desirable attributes are foods that are fresh, natural and minimally processed.” Chambers and Phan [3] showed that sensory aspects are the most important consideration in eating behavior, but health also is important in many food selections, which suggests that customers who say that “fresh” and “natural” are key considerations may be using those terms at least in part to refer to sensory and health properties. Other authors [4] have shown that using the term “natural” has an impact on liking or the hedonic sensory experience of products. Foods that are considered natural and not genetically modified had the most important attributes. The Neilson data [2] also found that labels that referenced ideas that consumers already had about the product were purchased more than other foods.

The appeal of naturalness is two-fold, with many people finding it healthier and more moral. Natural food is considered to be a more moral choice over artificial foods [5]. Bratanova et al. [6] found that moral food choices, through a sense of moral satisfaction, make food subjectively taste better, which serves as a reward mechanism for buying the product. Natural food also is considered to be healthier [5,7] with natural meat providing a mediational effect on the risk of colon cancer associated with meat consumption. Lay person comments on redefining natural submitted to the United States Food and Drug Adminisgtration (FDA) support the theory that natural is associated with healthful food [8].

The problem in reviewing naturalness is that what constitutes a natural food in scientific studies may be different among studies and may not be the same as the definition that consumers use [1]. Many studies have used the same criteria as “organic” food as their criteria for naturalness, while others have used the presence of some degree of natural ingredients to constitute naturalness. This variation in definitions causes a massive variation in results and muddies the waters of what exactly natural means for both data collection and marketing purposes for companies in the food business. The confusion is corroborated by consumer comments sent to the FDA [8] during the comment collection process, with the definitions given ranging from products that are “are healthy in some way” to anything that is “not fresh should not be considered healthy”. Similarly, Dominick et al. [9] found that 80% of respondents thought that “all natural” products would mean no antibiotics, no hormones, and no preservatives added in processing, with 60% of respondents sayings that natural food would have improved animal welfare practices, improved nutritional value, and improved food safety. A study in the Netherlands [10] found that those people who scored high on the scale of “prefers natural products” appeared to be most interested in “prevention-oriented motivations” (i.e., motivations for health and not eating foods they thought were unhealthful).

Other problems are that studies focus only on a single aspect to determine naturalness (for example, foods in packages [11]), or only on a single aspect of naturalness (e.g., health, environment, additives, etc.). In addition, some authors [12] have shown that natural may not be as holistically equivalent as others have suggested. For example, in a study in France, the respondents differentiated that health was not equivalent to natural, instead it was divided into a “natural” component associated with chemicals or GMOs (genetically modified organisms) and a component more related to disease that was not associated with natural.

The present research aims to investigate what percentage of the population considers various ingredients as natural, without giving participants a definition of natural. Such a procedure was used to best reflect what consumers believed was “natural”, without biasing them toward a particular point of view.

## 2. Materials and Methods

Qualtrics (Provo, UT, USA), an online survey company, was used to recruit 630 subjects nationwide, with proportions divided to represent the four demographic regions of the United States (U.S.) (Northeast, South, Midwest, and West), as defined by the U.S. Census Bureau. A gender quota was set (50% male and 50% female) for all the different age groups. The participants were divided into three age classes—(1) 18–34 years old; (2) 35–54 years old; and (3) more than 54 years old—and 100 respondents per demographic group (gender X age division). Additional information was obtained, such as education level and number of adults and children under the age of 18 in the household. The participants did not receive a financial incentive for completing the online survey, but the Qualtrics database has a reward system in order to compensate the respondents for their time and collaboration.

The questionnaire (see the sample in the Supplementary Material) was developed to evaluate consumer responses to whether a material was considered “natural” or not. A Check All That Apply format was used for the questionnaire, which simply required that the respondents check each ingredient they believed would qualify as “natural”. A total of 20 ingredients (Table 1) found in human foods in the U.S. was selected to represent a range of ingredients that consumers might find as common or unusual, common vs chemical names, and various sources and forms. Questions related to food and health beliefs (e.g., caring about ingredients, selecting GMO products, trying to eat for enjoyment) were also included to help determine the impacts of various health or food beliefs. The survey was conducted in English using Qualtrics survey software and was approved by the Institutional Review Board of Kansas State University.

The data were analyzed in Excel (Microsoft Office Pro ver. 2013) using descriptive statistics and chi-square tests for significance, considering a *p*-value under 5% to be significant.

## 3. Results

The results of the study found that corn, wheat flour, black beans, and soybeans were all considered natural by at least 60% of consumers (Table 2). Only those four foods, as well as sugar and salt, were perceived as natural by more than half of consumers. Xanthan gum, insect powder, maltodextrins, butylated hydroxyanisole (BHA), and sodium acid pyrophosphate (SAPP) were considered natural by the fewest consumers, with less than 10% of consumers considering those ingredients as natural.

Several inconsistencies were found in consumer opinions; most notable were the relationships between gluten and wheat flour, baking soda and sodium bicarbonate, and the differences between the various flours (pea, wheat, and sorghum). Wheat flour was considered natural by nearly three times as many people as gluten (a major protein in wheat flour). Baking soda also was more likely to be considered natural compared with sodium bicarbonate (the chemical name of baking soda). Interestingly, fewer than half as many people considered sorghum and pea flours natural as did wheat flour.

The data were cross tabulated between different demographic and belief factors asked during the survey. Age group showed the most significant (*p* < 0.05) differences; sorghum flour, black beans, lecithin, corn syrup, and molasses were all more likely to be considered natural by participants older than 55. Only insect powder and gluten were significantly (*p* < 0.05) less likely to be considered natural by those over 55 than other age groups. Gender was found to have a significant (*p* < 0.05) effect on three items—lecithin, corn syrup, and maltodextrins—with more men being more likely to consider each of those ingredients natural. Education was found to have no significant impact on perception of naturalness.

A number of demographic/attitudinal subgroups influenced just one or two ingredients. Those who responded that they agree with the statement “I care about the ingredients in my food” were significantly (*p* < 0.05) more likely to consider black beans and baking soda natural. People agreeing with the statement “I always follow a healthy diet” were more likely (*p* < 0.05) to consider both sorghum flour and sodium bicarbonate not natural. Respondents who agreed with the statement “I don’t worry about naturalness” were significantly (*p* < 0.05) less likely than expected to consider black beans and molasses natural. Those who stated “I look for non-GMO products” were not significantly different in their responses for any ingredient from those who did not choose that statement.

## 4. Discussion

The main results showed that no ingredient was determined to be natural by all people. In fact, no ingredient listed in the survey was found to be natural by more than 70% of U.S. consumers. This suggests that consumers have multiple reasons for not believing that certain ingredients are natural and, clearly, differ in the importance they place on various characteristics. This concept of complexity in consumer expectations is supported by the U.S. Federal Trade Commission who decided not to define natural in 1983 because, in part, “consumer expectations were too complex to address” [13].

We found it especially surprising that that corn and soybeans were considered to be natural by over 60% of consumers even though 92% of corn and 94% of soybeans are genetically modified in the United States [14]. There was also no statistical difference between those responding that they look for non-GMO products versus those who do not usually look for non-GMO products. Both products are highly marketed, familiar to consumers, and are considered healthy foods in general. The discrepancy between the two products is likely caused by the perceived healthiness and familiarity bias. However, some consumers definitely believe that products from corn, such as high fructose corn syrup, and the use of soy in some “natural” products are not appropriate, as noted by lawsuits over such products [13].

We note that items with chemical names tend to be characterized by almost all consumers as not natural. This is true even for ingredients that are manufactured, as well as those that are derived, from natural sources (e.g., xanthan gum). In addition, some ingredients, such as insect powder, which consumers may believe to be an adulterant or disgusting rather than a wholesome ingredient, cannot be natural. Shim et al. [15] found safety perception and knowledge of naturalness increased by 60% with communication programs that help customers become more familiar with the product.

The discrepancies noted in the results for items such as wheat flour vs gluten also have likely reasons. Those data suggest that particular portions of an ingredient or having to further process an ingredient to extract fractions (wheat flour vs gluten) or the perceived dietary constraints of an ingredient name (gluten) negatively impacts the perception of natural. Similarly, the use of a familiar vs chemical name (baking soda vs sodium bicarbonate) impacts the perception of natural, with the chemical name greatly reducing the perception of naturalness. The difference in flour types suggests that familiarity (wheat flour, which is a common ingredient, vs sorghum or pea flours, which are less common) plays a role in defining “natural”. Evans et al. [16] proposed the hypothesis that lesser known ingredients will be perceived to be less natural then well-known ingredients. Although these reasons may seem obvious or valid on the surface to both researchers and consumers, they also clearly show the difficulty in defining what is natural. If wheat flour is considered natural (63% of consumers said it was), but sorghum flour, which is more likely to be GMO-free and perhaps more likely to be organic, is not considered natural (only 23% of consumers thought so), what then constitutes reality? A clear definition could help in labeling, but getting to that point may pose huge problems because of the disconnect between science and the consumer’s perception or knowledge [13].

We believe that the familiarity bias explains why men were more likely than women (*p* <0.05) to respond that maltodextrins and lecithin were natural. Both products are frequently used in protein powders and protein snacks, which tend to be marketed and consumed more by men. The clearest evidence of the familiarity bias is the difference in those saying that baking soda was natural compared with those who said that sodium bicarbonate was natural. As shown in several studies, chemical names or “e numbers” (used in Europe on ingredient labels) [7] tend to be considered less natural by consumers.

Insect powder and pea flour appear to be good examples of food neophobia affecting the perceived naturalness of ingredients. An individual’s expectations toward food products play a critical role in consumer psychology [17], and flours and powders are not what U.S. consumers’ think of when they think of insects and peas. There may be a neophobic response to their acceptance, which will influence the perception of naturalness. It is likely that this effect is amplified by the fact that insect powder and pea flour are “novelty” foods, to which most Americans have had little exposure. It also is likely that the physical change (manufacturing) of turning the ingredient into a powder is driving down the perception of naturalness. Rozin [18] found that grinding peanuts into peanut butter reduced overall perceptions of naturalness by 8.3%. 

The fact that those over 55 years of age were more likely to consider a product natural is interesting. As Walker et al. [19] found in 1991, the biggest risk factors for dietary inadequacies were loneliness and social isolation. However, in recent years, technology and culture changes in the United States have mitigated these issues, because those over 55 now have more access to the internet, social programming and healthcare services, and nutritional information. With increasing life expectancy, older adults are taking a bigger role in their own health. The only ingredient for which the results were lower than expected for that age group was gluten, and we expect that this was because of the gluten scare that has occurred over the last few years. This, directly refutes claims made by Rozin et al. [18] that age and other demographic factors do not contribute much to the perception of naturalness and suggests that changes in the market in the last 5–6 years may be changing the perceptions of some consumers.

We believe that lack of familiarity explains the low percentages for ingredients such as molasses and sorghum. The decline in the popularity of molasses in the U.S. after World War II and the fact that few human foods contain grain sorghum (in the past, it was used primarily for animal feed in the U.S.) means that both products are not widely known, and thus, people do not say that they are natural.

The results also indicate an ideological gap between “what people think constitutes a natural product” and “what products are natural”. As discussed by Román et al. [1] studies about naturalness usually research what factors make a product natural and how product manipulation affects naturalness. Instead, our study focuses on the consumers’ perception of a holistic ingredient. Thus, rather than examining ingredients in the context of whether certain aspects of an ingredient are natural, our study examined ingredients as a single concept. Thus, an ingredient may not be considered natural when considered against a particular criterion but could be considered natural by consumers simply have an image of a product (in this case an ingredient) in their minds outside of the context of a specific set of values or motivations.

One limitation of this study is that consumers were not asked to explain why they considered a food natural or not. The survey provides only an understanding of the likelihood that an ingredient is perceived as natural. Thus, we can speculate, but not cannot be sure of the reasoning of consumers for their responses. Further research is needed for such information.

## 5. Conclusions

Our results show that consumers do not agree on which ingredients are natural and which are not, with a few exceptions. Thus, it is likely that consumers have different definitions for what constitutes natural. This allows for greater consumer confusion and frustration when trying to find and purchase natural food products and ingredients. We believe that that further research is needed to help determine what consumers believe constitutes natural. Moreover, we think that there is a lack of research into what ingredients people consider natural, not just what makes a food natural. Further research in this area, including in other countries and to determine the reasons people believe various ingredients may not be natural, is necessary.

One key aspect that is lacking appears to be agreement in the scientific community and a coherent educational campaign as to “what is natural”. Research already shows that disagreement exists at the product (food or beverage) level. If further research shows that even at the ingredient level consumers cannot agree on what constitutes natural in part because there is confusion over what the term means, then educational efforts to explain the term are needed. In addition, better consumer information about some ingredients (e.g., sorghum) or new products is needed in order to overcome what we believe is a lack of familiarity that can breed unease with consumers.

## Figures and Tables

**Table 1:** Demographics Percentages *.

Demographic Characteristics	Variables	Percentage
Gender	Men	50%
	Women	50%
Age	18–34	33%
	35–54	33%
	≥55	33%
Level of Education	Primary School or Less	1%
	High School	45%
	College or University	53%
Number of Adults Living at Residence	1	27%
	2	48%
	3	15%
	4	7%
	5 or More	3%
Number of Children Living at Residence	0	67%
	1	16%
	2	10%
	3	4%
	4 or More	2%

* Percentages were based on 630 consumers in total.

**Table 2:** Percentage of people saying that an ingredient was natural.

Ingredients	Percent Saying Ingredient Was Natural
Corn	69%
Wheat Flour	63%
Black Beans	63%
Soybeans	62%
Salt	54%
Sugar	54%
Molasses	45%
Canola Oil	42%
Pea Flour	38%
Baking Soda	33%
Sorghum Flour	24%
Gluten	23%
Corn Syrup	20%
Lecithin	14%
Sodium Bicarbonate	13%
Xantham Gum	8%
Insect Powder	7%
Maltodextrins	5%
Butylated Hydroxyanisole (BHA)	4%
Sodium Acid Phyrophosphate (SAPP)	3%

## Supplementary Materials

The following are available online at http://www.mdpi.com/2304-8158/7/4/65/s1. Supplementary Material: Natural Portion of U.S. Survey.

## References

[1] Román S., Sánchez-Siles L.M., Siegrist M. (2017). The importance of food naturalness for consumers: Results of a systematic review. Trends Food Sci. Technol..

[2] Nielsen (2015). We Are What We Eat Healthy Eating Trends around The World.

[3] Phan U.X.T., Chambers IV E. (2016). Application of an eating motivation survey to study eating occasions. J. Sens. Stud..

[4] Apaolaza V., Hartmann P., López C., Barrutia J.M., Echebarria C. (2014). Natural ingredients claim’s halo effect on hedonic sensory experiences of perfumes. Food Qual. Pref..

[5] Rozin P., Spranca M., Krieger Z., Neuhaus R., Surillo D., Swerdlin A., Wood W. (2004). Preference for natural: Instrumental and ideational/moral motivations, and the contrast between foods and medicines. Appetite.

[6] Bratanova B., Vauclair C., Kervyn N., Schumann S., Wood R., Klein O. (2015). Savouring morality. Moral satisfaction renders food of ethical origin subjectively tastier. Appetite.

[7] Siegrist M., Sütterlin B. (2017). Importance of perceived naturalness for acceptance of food additives and cultured meat. Appetite.

[8] Museum of Food and Drink Questionnaire with Multiple Responses. US Food and Drug Administration 2016. https://www.regulations.gov/document?D=FDA-2014-N-1207-7334.

[9] Dominick S.R., Fullerton C., Widmar C.J.O., Wang H. (2017). Consumer Associations with the “All Natural” Food Label. J. Food Prod. Mark..

[10] De Boer J., Schösler H. (2016). Food and value motivation: Linking consumer affinities to different types of food products. Appetite.

[11] Binninger A.-S. (2017). Perception of naturalness of food packaging and its role in consumer product evaluation. J. Food Prod. Mark..

[12] Sautron V., Péneau S., Camilleri G.M., Muller L., Ruffieux B., Hercberg S., Méjean C. (2015). Validity of a questionnaire measuring motives for choosing foods including sustainable concerns. Appetite.

[13] Parasidis E., Hooker N., Simons D.T. (2015). Addressing consumer confusion surrounding “natural” food claims. Am. J. Law Med..

[14] U.S. Department of Agriculture Economic Research Service Adoption of Genetically Engineered Crops in the U.S. USDA 2017. https://www.ers.usda.gov/data-products/adoption-of-genetically-engineered-crops-in-the-us.aspx.

[15] Shim S.M., Seo S.H., Lee Y., Moon G.I., Kim M.S., Park J.H. (2011). Consumers’ knowledge and safety perceptions of food additives: Evaluation on the effectiveness of transmitting information on preservatives. Food Control.

[16] Evans G., de Challemaison B., Cox D.N. (2010). Consumers’ ratings of the natural and unnatural qualities of foods. Appetite.

[17] Tuorila H., Meiselman H.L., Bell R., Cardella A.V., Johnson W. (1994). Role of sensory and cognitive information in the enhancement of certainty and liking for novel and familiar foods. Appetite.

[18] Rozin P., Fischler C., Shields-Argelès C. (2012). European and American perspectives on the meaning of natural. Appetite.

[19] Walker D., Beauchene R.E. (1991). The relationship of loneliness, social isolation, and physical health to dietary adequacy of independently living elderly. J. Am. Diet. Assoc..

