# Supplementary material for: What Is “Natural”? Consumer Responses to Selected Ingredients
Source: Foods. 2018 Apr 23;7(4):65. doi: 10.3390/foods7040065 (PMC5920430; doi:10.3390/foods7040065)
Supplement: Supplementary file 1 [file foods-07-00065-s001.pdf]

Natural Portion of U.S. Survey

D1 What is your gender?

- ○ Male (1)
- ○ Female (2)

D2 What is your age?

- ○ 18 - 34 (1)
- ○ 35 - 54 (2)
- ○ 55 + (3)

D3 What is the highest degree you completed?

- ○ Primary School or less (1)
- ○ High School (2)
- ○ College or University (3)

Q1 I care about the ingredients in my food.

- Strongly Disagree (1)
- Disagree (2)
- Disagree somewhat (3)
- Neither agree nor disagree (4)
- Agree somewhat (5)
- Agree (6)
- Strongly Agree (7)

Page Break

Q2 I look for only Non-GMO (Non-Genetically Modified Organisms) ingredients on the food I eat.

- Strongly Disagree (1)
- Disagree (2)
- Disagree somewhat (3)
- Neither agree nor disagree (4)
- Agree somewhat (5)
- Agree (6)
- Strongly Agree (7)

Page Break

Q3 I do not read the ingredient statements and don’t worry about natural ingredients.

- Strongly Disagree (1)
- Disagree (2)
- Disagree somewhat (3)
- Neither agree nor disagree (4)
- Agree somewhat (5)
- Agree (6)
- Strongly Agree (7)

Page Break

Q14 I always follow a healthy and balanced diet.

- Strongly Disagree (1)
- Disagree (2)
- Disagree somewhat (3)
- Neither agree nor disagree (4)
- Agree somewhat (5)
- Agree (6)
- Strongly Agree (7)

Q. Please check each ingredient that you **would consider “Natural"**. (note: the numbers after the ingredients were used simply for randomization on each questionnaire and did not appear on the questionnaire)

- ☐ Wheat flour (1)
- ☐ Sorghum flour (2)
- ☐ Soybean (3)
- ☐ Sodium Bicarbonate (4)
- ☐ Corn (5)
- ☐ Salt (6)
- ☐ BHA (Butylated hydroxyanisole) (7)
- ☐ Gluten (8)
- ☐ SAPP (Sodium Acid Pyrophosphate) (9)
- ☐ Insect powder (10)
- ☐ Xanthan Gum (11)
- ☐ Pea flour (12)
- ☐ Canola Oil (13)
- ☐ Black beans (14)
- ☐ Baking Soda (15)
- ☐ Lecithin (16)
- ☐ Corn Syrup (17)
- ☐ Sugar (18)

- [ ] Molasses (19)
- [ ] Maltodextrins (20)

Page Break
